# Non-islet Cell Hypoglycemia: Case Series and Review of the Literature

**DOI:** 10.3389/fendo.2019.00316

**Published:** 2019-05-15

**Authors:** Vishnu Garla, Hardik Sonani, Venkatraman Palabindala, Celso Gomez-Sanchez, Jose Subauste, Lillian Francis Lien

**Affiliations:** ^1^Department of Internal Medicine, University of Mississippi Medical Center, Jackson, MS, United States; ^2^Icahn School of Medicine at Mount Sinai, New York, NY, United States

**Keywords:** non-islet cell hypoglycemia, hypoglycemia, hepatocellular carcinoma, gastrointestinal stromal tumor, IGF-2 induced hypoglycemia

## Abstract

Non-islet cell hypoglycemia (NICH) is hypoglycemia due to the overproduction of insulin-like growth factor-2 (IGF-2) and its precursors which can activate the insulin receptor. Typically, large mesenchymal and epithelial tumors can cause NICH. Diagnosis is confirmed by finding an elevated IGF-2/IGF-1 ratio. The mainstay of treatment is surgical excision. Glucocorticoids may be used in cases where surgery is not possible. We present two cases of NICH with different outcomes. A 33-year-old male patient admitted with altered mental. He was found walking naked outside his house. Laboratory assessment revealed severe hypoglycemia. Further evaluation showed low levels of insulin, C-peptide, and beta-hydroxybutyrate along with an elevated IGF-2/IGF-1 ratio confirming the diagnosis of NICH. Computed tomography (CT) of the abdomen showed a massive tumor of the liver consistent with hepatocellular carcinoma. Since the patient refused surgery, he was started on prednisone however the hypoglycemia persisted. A 54-year-old female patient with a history of type 2 diabetes mellitus (DM) admitted with recent onset hypoglycemia. Despite stopping her insulin, she continued to have hypoglycemia necessitating the administration of high concentrations of intravenous dextrose. Further evaluation showed low levels of insulin, C-peptide, and beta-hydroxybutyrate along with an elevated IGF-2/IGF-1 ratio consistent with the diagnosis of NICH. CT abdomen showed a 24 cm tumor near the uterus. The pathology was consistent with a gastrointestinal stromal tumor (GIST). After surgical excision of the tumor, the hypoglycemia resolved.

## Introduction

Non-islet cell hypoglycemia (NICH) is a rare cause of hypoglycemia which is due to excessive secretion of insulin-like growth factor (IGF)-2 or pro IGF-2. These molecules can activate the insulin receptor and can cause hypoglycemia. NICH is seen in association with epithelial and mesenchymal tumors. (1) NICH is potentially misdiagnosed or underdiagnosed due to its rarity, non-classical clinical presentation, and an ambigous lab picture. It can cause profound and persistent hypoglycemia until the surgical excision of the tumor is performed, which can potentially influence the management strategy for the treatment of cancer (2).

We report two cases of NICH one secondary to hepatocellular carcinoma and the other due to a gastrointestinal stromal tumor (GIST).

## Case 1

**Figure 1:**
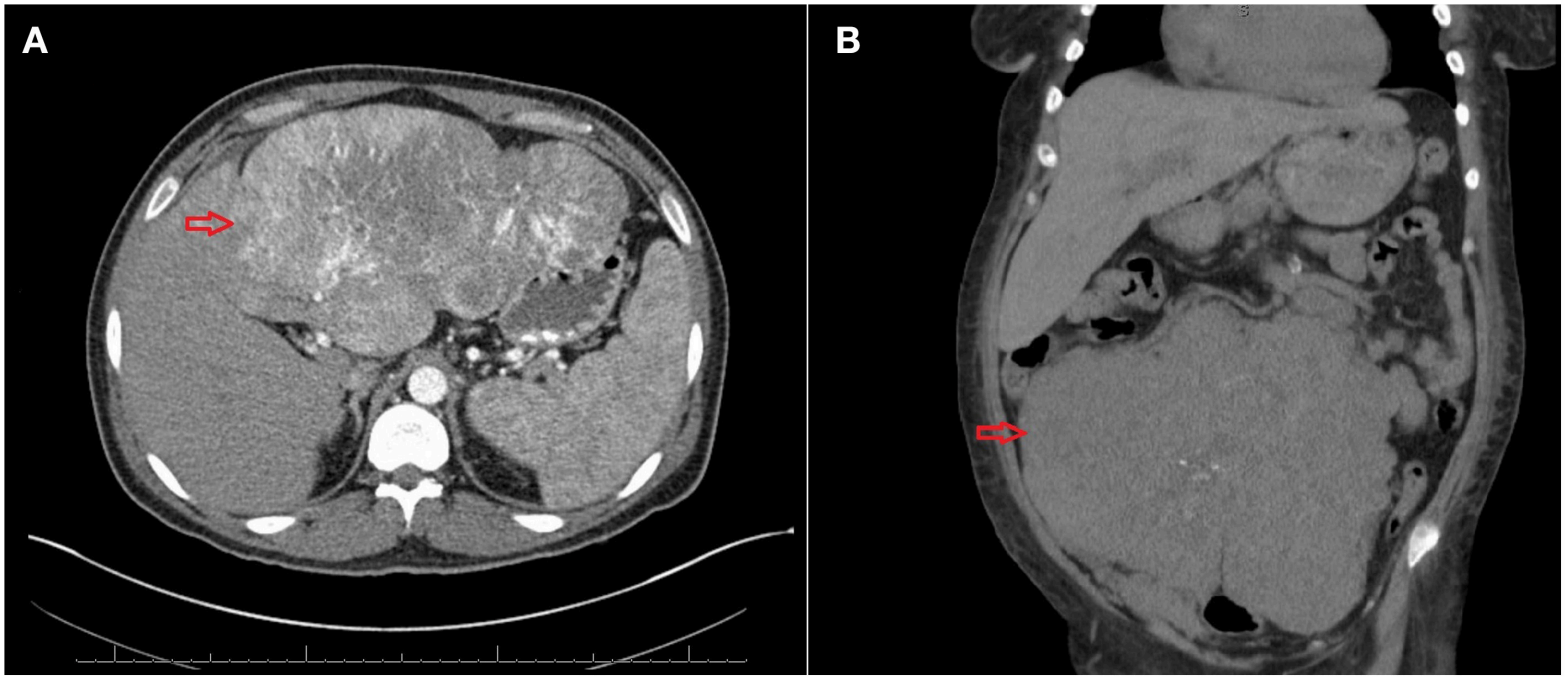
**(A)** Computed tomography (CT) of the abdomen showing a large mass of 20 cm size (red arrow). **(B)** CT of the abdomen showing a large tumor of 24 cm^*^ 18 cm^*^12 cm (red arrow).

**Figure 2:**
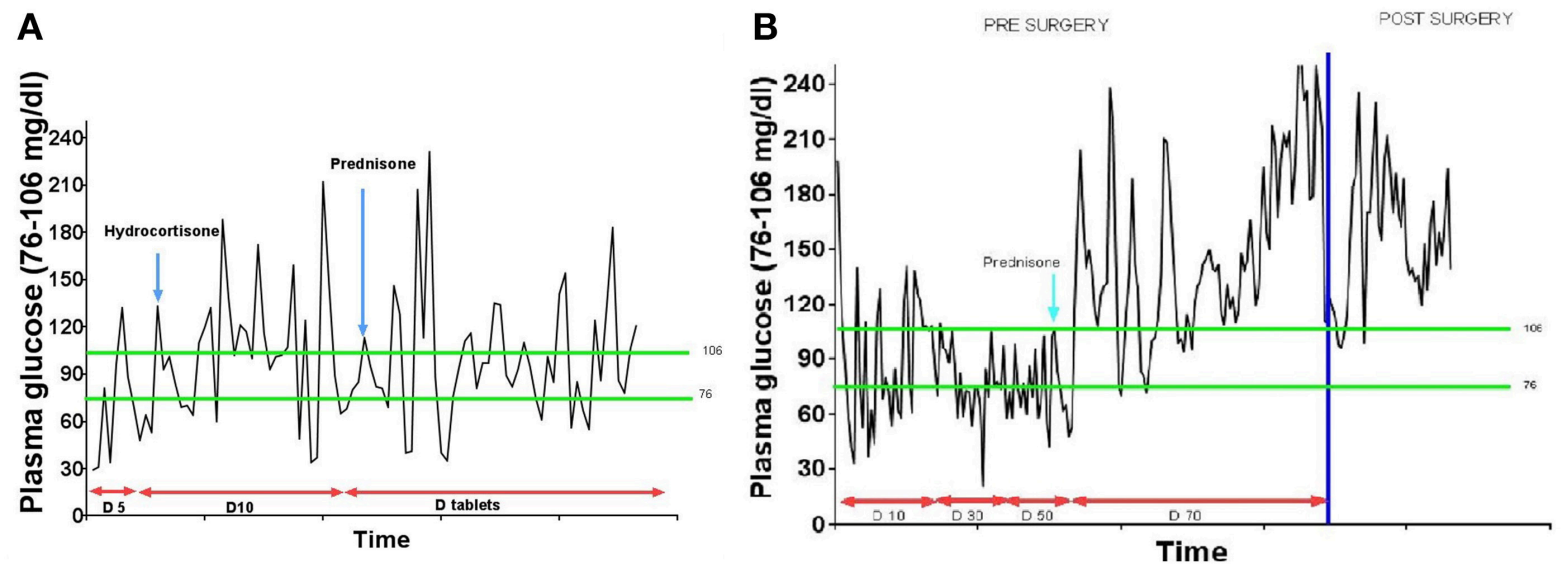
**(A)** Plasma glucose trend in case 1. **(B)** Plasma glucose trend in case 2.

A 33-year-old Hispanic male who was brought to the emergency room with altered mental status. He was found by his coworkers to be incoherent and was walking naked outside his home. Blood glucose was low at 29 (74–106 mg/dl). He had persistent hypoglycemia despite receiving several ampoules of 50% dextrose. A 5% dextrose infusion was started which maintained glucose in the normal range. The patient did have some improvement in his mental status but not complete normalization. He denied any history of diabetes mellitus, alcohol abuse, or illegal drug use. History was significant for a 30lb weight loss over the last 4 months. The physical examination was significant for a stellate laceration in the occipital region and marked hepatomegaly. He was oriented to time, place and person. Ultrasound of the abdomen revealed a 15 cm mass in the left lobe of the liver concerning for malignancy. Elevated alpha-fetoprotein and liver biopsy both were consistent with the diagnosis of hepatocellular cancer. Further laboratory assessment for the evaluation of hypoglycemia showed low insulin, c peptide, proinsulin, and beta-hydroxybutyrate. Insulin antibodies and sulfonylurea screen were negative. Insulin-like growth factor 2 (IGF-2) was normal; however, the insulin-like growth factor-1 (IGF-1) was suppressed. The IGF-2/IGF-1 ratio was >10, consistent with the diagnosis of NICH (Table 1). Computed tomography (CT) of the chest and abdomen showed a large mass of 20 cm size in the liver and a solitary nodule in the right lung which was consistent with metastasis (Figure 1). Since the patient continued to be hypoglycemic, he was started on glucocorticoids (initially hydrocortisone and then prednisone to a maximum dose of 40 mg). Despite this, the patient continued to have hypoglycemic episodes (Figure 2). A bone scan revealed further metastasis in the right clavicle and scapula. Palliative debulking of the tumor was considered however was deferred per the patient's wishes.

**Table 1:** Laboratory assessment of hypoglycemia.

	**Case 1**	**Case 2**
Plasma glucose (74–106 mg/dl)	34	54
Insulin (2–25 μU/l)	< 1	< 1
C peptide (0.78–5.19 ng/ml)	0.04	0.25
Proinsulin (3–20 pmol/l)	2.3	2.9
Beta hydroxybutyrate (0.2–2.81 mg/dl)	0.71	0.35
Insulin antibodies (0.00–0.02 nmol/ml)	0	0
Sulfonylurea screen	Negative	Negative
IGF-1 (108–167 ng/ml)	23	54
IGF-2 (288–736 ng/ml)	506	609
IGF-2/IGF-1 ratio	22	11.27

## Case 2

**Figure 3:**
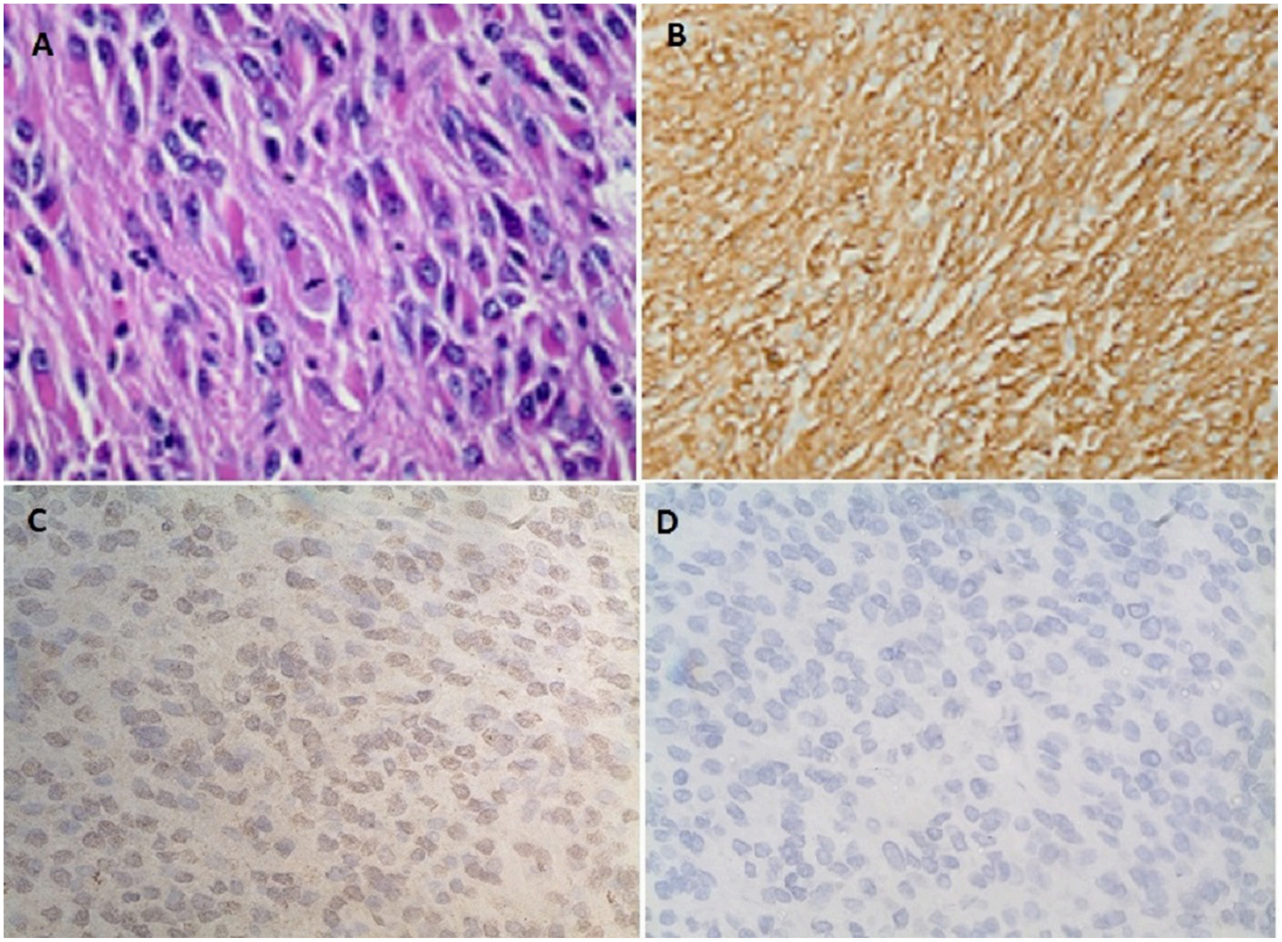
**(A)** Spindle cells with fibrillary eosinophilic cytoplasm forming whorls consistent with gastrointestinal stromal tumor (GIST). **(B)** Diffuse positive staining to CD 117. **(C)** Immunohistochemistry showing diffuse positive staining to IGF-2 monoclonal antibody (MA5-17096, Thermofisher scientific Inc.). **(D)** No positive staining seen on the control for IGF-2 antibody (MA5-17096, Thermofisher scientific Inc.).

A 54-year-old African American female patient who was transferred to our hospital for evaluation of hypoglycemia. Her past medical history was significant for diabetes mellitus on 10 units glargine nightly, end-stage renal disease on hemodialysis, hypertension, and uterine fibroids. Before the admission, she had hypoglycemic episodes for the last 2 weeks which persisted even after she stopped taking her insulin. These hypoglycemic episodes were characterized by sweating, anxiety, and confusion. A 20% dextrose infusion, but her hypoglycemia persisted. Initially, inadvertent intake of insulin or persistence of insulin due to renal failure were thought to be the cause of hypoglycemia. However, low plasma insulin, proinsulin, c peptide, and beta-hydroxybutyrate all pointed toward hypoglycemia secondary to a non-islet cell tumor (Table 1). IGF-1 was suppressed with a normal IGF-2 however the molar ratio was high confirming the diagnosis of IGF-2 induced hypoglycemia. CT abdomen revealed a 24 cm mass adjacent to the uterus (Figure 1). Hypoglycemia was persistent and necessitated the use of high concentration of dextrose (D) solution (up to D 70%) and multiple administration of rescue D 50% ampoules (Figure 2). A core biopsy was performed, and the pathology was consistent with a gastrointestinal stromal tumor (GIST). During exploratory laparotomy, a large vascular mass adherent to the sigmoid colon was observed. Excision of the mass along with a sigmoid colectomy with a colostomy, hysterectomy, oophorectomy, and an appendectomy was performed. Postoperatively she was hyperglycemic, and the dextrose infusion was discontinued. Pathology confirmed the diagnosis of GIST of the small intestine which stained positively for IGF-2 (Figure 3). Upon follow up in the endocrine clinic after 2 months she was euglycemic on her original home insulin regimen.

## Discussion

Hypoglycemia can be secondary to diverse etiologies; however, it is most often seen in diabetic patients on insulin or insulin secretagogues. NICH is a rare cause of hypoglycemia due to excessive secretion of IGF-2 or pro-IGF-2 (3, 4). NICH was first described in 1929 in a patient with hepatocellular carcinoma (5). Hypoglycemia was believed to be due to excessive consumption of glucose by the tumor until Unger et al. (6) suggested it was secondary to a humoral factor. Megyesi et al. demonstrated the presence of IGF in the tumor and also the resolution of hypoglycemia after excision of the tumor (7). Rinerkencht isolated IGF-1 and IGF-2 in 1978 while Dughaday et al. described NICH with normal IGF-2 but high pro-IGF-2 levels (8, 9).

NICH is mostly seen in mesenchymal and epithelial tumors. The most common tumor to be associated with NICH is hepatocellular carcinoma. However, the list of tumors associated with NICH is steadily increasing (Table 2). Most of these tumors are large; Both, benign, and malignant tumors have been associated with NICH (4). With the increasing incidence of hepatocellular cancer, the incidence of NICH is expected to increase but has remained steady, likely secondary to under diagnosis (10).

**Table 2:** Tumors associated with non-islet cell hypoglycemia.

Hepatocellular carcinoma
Fibrosarcoma
Mesothelioma
Adrenocortical carcinoma
Hemangiopericytoma
Stomach carcinoma
Pancreatic carcinoma
Medullary thyroid carcinoma
Lymphoma/Leukemia
Carcinoid syndrome

Typically in patients with IGF-2 induced hypoglycemia, the levels of insulin, and IGF-1 are low whereas the level of IGF-2 can be normal or elevated (11). For a long time the mechanism by which a normal IGF-2 could cause hypoglycemia remained a mystery, till the identification of an incompletely processed pro-IGF-2 (aka big IGF-2) in 1988 (9). IGF-2omas can secrete excessive quantities of pro-IGF-2 due to loss of imprinting secondary to activation of abnormal promoters. This relative excess of pro-IGF-2 may overwhelm the enzymes which usually process pro IGF-2 to mature IGF-2 (11–13).

Hypoglycemia may be the presenting symptom in approximately half the patients with IGF-2 producing tumors. In other cases, the diagnosis of cancer may precede the hypoglycemia (14, 15). Typically hypoglycemia is seen in the fasting state. Due to the repeated attacks of hypoglycemia, neuroglycopenic symptoms can predominate. Confusion, psychosis, amnesia, and seizures can be the presenting symptoms of NICH. Our first patient presented with confusion and amnesia likely due to the repeated attacks of hypoglycemia (16). NICH needs to be considered along with opioid use, cerebral metastasis, and infections in any cancer patient with altered mental status. NICH tends to have more severe symptoms than fasting hypoglycemia at the same glucose level due to the lack of ketogenesis secondary to the activation of the insulin receptor (IR) by IGF-2 (17). Acromegaloid changes secondary to the activation of IGF receptors by IGF-2 have been described in rare instances. Trivedi et al. reported a patient with pelvic clear cell sarcoma who developed acromegaloid changes which resolved after excision of the tumor (18).

**Figure 4:**
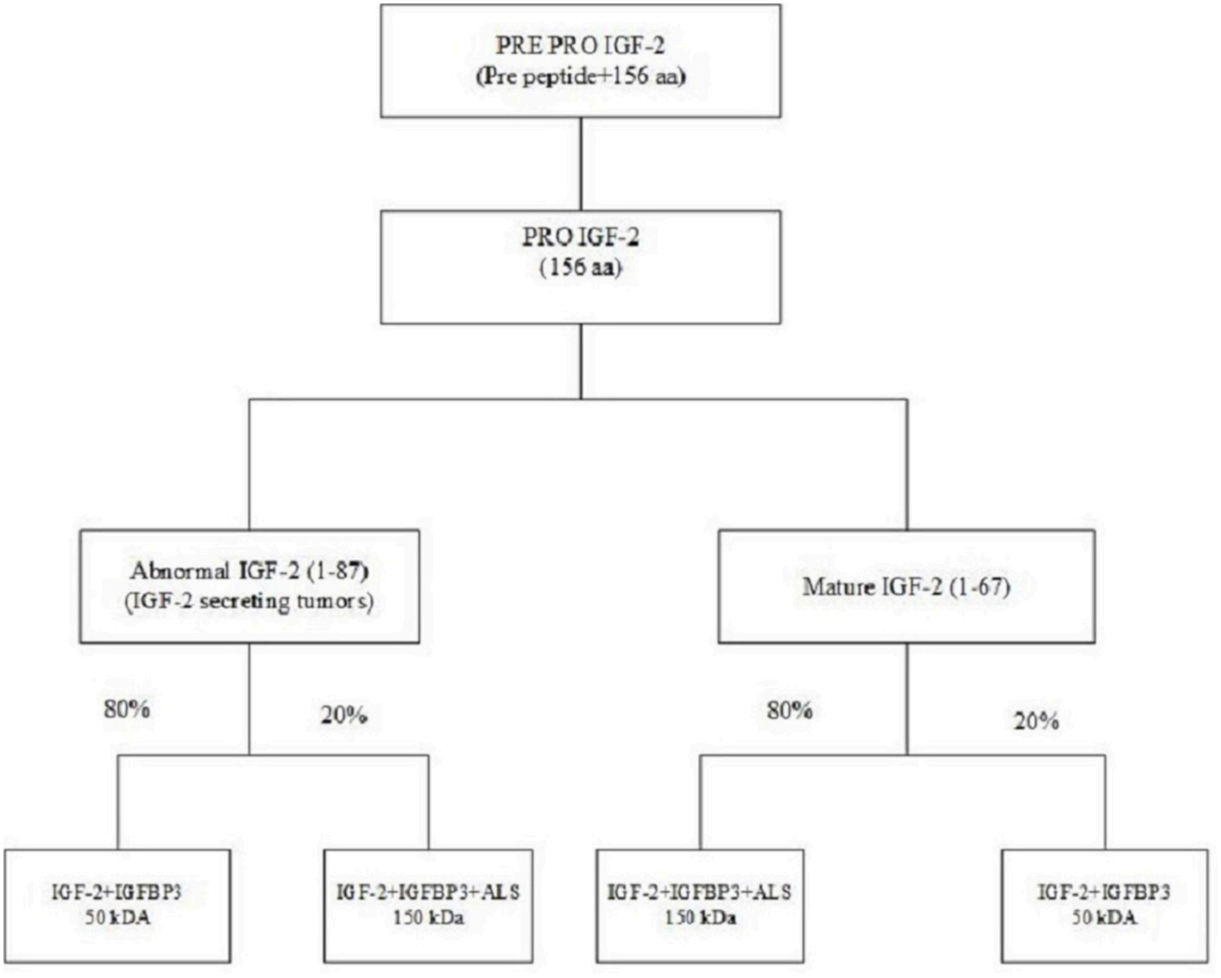
Synthesis and binding of mature and abnormal IGF-2.

NICH is secondary to excessive secretion of IGF-2 or pro-IGF-2. While IGF-2's role in fetal development is well-known, but its role in adults is less well-defined (19, 20). The IGF-2 gene is located on the short arm of chromosome 11 between the insulin and H19 genes (21). This gene is translated into a pre-pro IGF-2 peptide which consists of a 24 amino acid N terminal, 67 amino acid mature IGF-2, and an 89 amino acid C terminal. The pre-pro IGF-2 undergoes post-translational modification by removal of the N terminal, the addition of sialic acid oligosaccharides to the E domain and subsequent proteolysis of the E domain giving rise to various pro-IGF-2. Prohormone convertase converts pro IGF-2 to mature IGF-2 (67 amino acids); however, in IGF-2 secreting tumors, an abnormal IGF-2 (87 amino acids) is produced (22). IGF-2 is bound in the circulation to IGFBP-3. About 70–80% of IGF-2 is transported in the form of a 150 kDa complex consisting of IGF-2, IGFBP-3 and acid-labile subunit (ALS), and 20% in a 50 k-Da binary complex of IGF-2 and IGFBP-3. In IGF-2 producing tumors the ratio of the 150 and 50 kDa subunits is reversed. The 50 kDa subunits which are overproduced in NICH have greater biological activity and also can cross the capillary membranes to interact with insulin receptors (Figure 4) (23, 24).

There are two isoforms of insulin receptor (IR) which are formed due to alternate splicing IR-A and IR-B. IGF-2 can bind with high affinity to both the IGF-1 receptor and insulin receptor-A (IR-A) and with low affinity to insulin receptor-B (IR-B). The concentration of a particular isoform of IR is tissue dependent. IR-A expressed more in malignancies and embryonic tissues while IR-B which mediates the metabolic effects of insulin is expressed more in the liver, muscle, and adipose tissue. There are heterologous receptors formed due to heterodimerization of IGF-1 receptor and IR. Therefore, the symptoms of NICH depend on not only the level of IGF-2 but also the available concentration of receptors (4).

**Figure 5:**
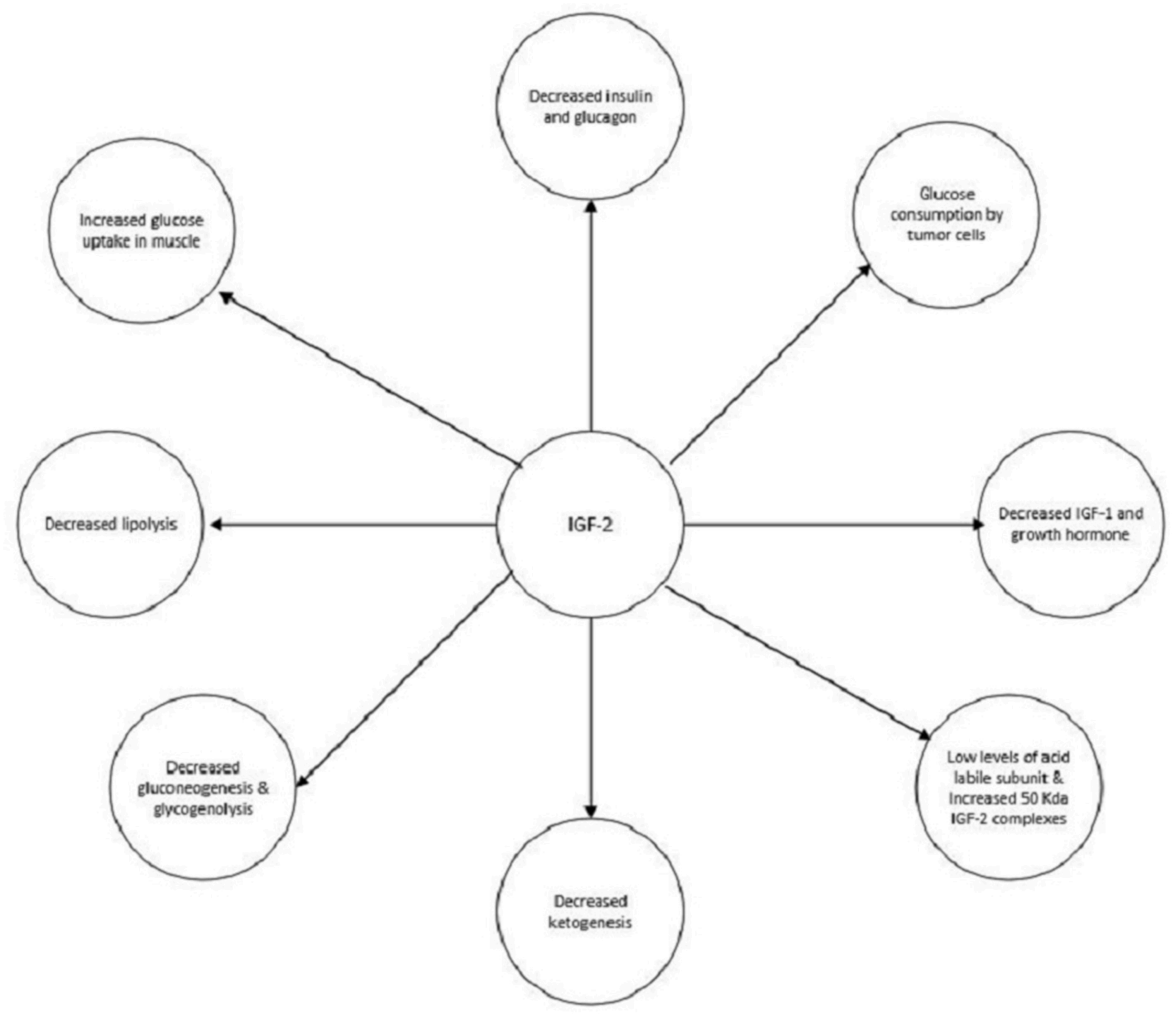
Mechanisms by which IGF-2 induced hypoglycemia.

IGF-2 has multiple actions which can contribute to the development of hypoglycemia (Figure 5). The main mechanism is shutting down hepatic glucose output. Similar to insulin, IGF-2, through activation of the insulin receptor, can inhibit gluconeogenesis, glycogenolysis, and ketogenesis. These actions are mediated through the action on insulin receptors on the hepatocytes and in the hypothalamus which communicate with the liver via the vagal nerve (25–27). IGF-2 also increases the uptake of glucose by muscles and inhibits lipolysis by activating the IR (28, 29). Also, growth hormone and IGF-1 levels are suppressed via the activation of IGF-1 receptors in the hypothalamus by IGF-2. Glucagon production is suppressed as well due to the activation of IGF-1 receptors on the pancreatic alpha cells by IGF-2 (30, 31). Although glucose consumption by the tumors could contribute to hypoglycemia this does not appear to be a significant pathway (4).

The initial step in the evaluation of hypoglycemia is documenting all components of Whipple's triad, i.e., the presence of signs and symptoms of hypoglycemia, a low plasma blood glucose, and resolution of the symptoms with administration of glucose. A detailed history to evaluate for any systemic diseases or medications which can predispose to hypoglycemia is essential (32).

In order to delineate the etiology of hypoglycemia plasma insulin, C-peptide, proinsulin, and beta-hydroxybutyrate must be drawn when the patient is hypoglycemic. NICH presents with low levels of insulin, C-peptide, proinsulin, and beta-hydroxybutyrate (Table 3) (32). Further evaluation of NICH consists of measuring the IGF-1 and IGF-2 levels. IGF-1 is typically suppressed while the levels of IGF-2 may be normal or elevated. An IGF-2/IGF-1 molar ratio of >10 confirms NICH. However, it must be kept in mind that IGF-2/IGF-1 ratio may be >10 in malnutrition and sepsis; however, in these cases both IGF-2 and IGF-1 are low. There is no commercially available assay for pro-IGF-2 (33, 34). It must also be noted that the IGF-2/IGF-1 ratio is influenced by the level of IGFBP-3. Therefore, conditions, where the level of IGFBP-3 is reduced like in renal failure, may have a false negative IGF-2/IGF-1 ratio (4).

**Table 3:** Differential diagnosis of non-islet cell hypoglycemia.

	**Insulin (3 mcu/ml)**	**C-peptide (0.2 mmol/L)**	**Proinsulin 5 pmol/L**	**Beta-hydroxybutyrate (2.7 mmol/L)**
Insulinoma/sulfonylurea	High	High	High	Low
Exogenous insulin	High	Low	Low	Low
Nonislet cell hypoglycemia	Low	Low	Low	Low
Insulin-independent hypoglycemia	Low	Low	Low	High

*mcu, microunits; mmol, millimole; pmol, picomoles; L, liter; ml, milliliter*.

Surgical excision of the tumor is the most definitive treatment of NICH. It results in an immediate resolution of hypoglycemia as seen in Case 2. Debulking should be considered where complete surgical excision is not feasible (2, 35, 36). De Boer et al. describes a case of successful treatment of NICH with neoadjuvant chemoradiation along with embolization of the feeding vessels in a patient with a non-resectable solitary fibrous tumor (37).

Glucocorticoids may be used in the treatment of NICH. They prevent hypoglycemia by increasing hepatic gluconeogenesis, inhibiting peripheral uptake of glucose, and promoting lipolysis (Figure 5).The dose needs to be titrated to manage the hypoglycemia appropriately. As seen in both of our cases, the hypoglycemia resolved to an extent once glucocorticoids administration was started (2). Glucagon increases glycogenolysis and gluconeogenesis and can prevent hypoglycemia. Hoff et al. have used glucagon infusions via a pump to treat NICH in a patient with meningeal sarcoma. However, effects only last as long as glucagon is administered; therefore it is only a short term treatment (38). Recombinant growth hormone (rGH) by increasing gluconeogenesis and peripheral glucose uptake can alleviate hypoglycemia. They also alter the production of IGF-2 by increasing the 150 kDa complexes and decreasing the 50 kDa complexes. The former is much less efficacious at producing hypoglycemia. rGH has been used in cases of NICH which were unresponsive to glucocorticoids (39).

As NICH results from the interaction of big IGF-2 and the insulin receptor, therapies aimed at disrupting this interaction would potentially be useful in the treatment of NICH. Prince et al. have developed a high-affinity antibody to IGF-2 which comprises of human IgG1 Fc domain and a modified domain-11 of the IGF-2 receptor (which binds specifically to IGF-2). Substitution of glutamic acid to lysine in the 1,554 position (IGF-2Re1554k-Fc protein) further enhances the specificity and affinity for binding to IGF-2 (40). Feng et al. have developed an antibody (IgG1 m610) which has specificity for both mature and pro IGF-2 (41). Other approaches include enhancing prohormone convertase (which converts pro to mature IGF-2) and use of anti-IGF-2 small interfering RNA (42, 43).

## Conclusion

NICH is a rare cause of hypoglycemia however it is one which is likely to be underdiagnosed Physicians need to be vigilant about NICH, as it is increasingly seen, given the increased survival of hepatitis and hepatocellular carcinoma patients. It is also being reported in association with an ever increasing number of cancers. NICH can cause severe hypoglycemia and may present with predominantly neuroglycopenic symptoms. Although altered mental status in a cancer patient could be due to narcotic use, brain metastasis or CNS infections, NICH needs to be in the differential diagnosis.

NICH is characterized by low levels of insulin, C-peptide, proinsulin, and beta-hydroxybutyrate. A molar ratio of IGF-2/IGF-1 higher than 10 confirms NICH. Definitive surgery may have to be done for the alleviation of hypoglycemia even though it may not influence the prognosis of cancer. If complete excision is not possible, debulking or embolization should be considered. Glucocorticoids are the preferred medical modality for treatment of NICH however hypoglycemia recurs once they are stopped, without further definitive treatment.

Further research is needed as to why only a few patients develop NICH, or to establish if there is a critical mass beyond which NICH is likely to occur. Studies are also needed to study the efficacy of newer antibodies.

## Ethics Statement

This was exempt from ethics committee approval as it is a case report. Written and informed consent was obtained from the patients to submit their cases to the journal.

## Author Contributions

VG, HS, and VP were involved in writing the case descriptions and discussion section of the manuscript. CG-S was involved in doing the antibody staining, obtaining pictures and graphs, and also editing the manuscript. JS and LL were involved in editing the manuscript.

### Conflict of Interest Statement

The authors declare that the research was conducted in the absence of any commercial or financial relationships that could be construed as a potential conflict of interest.
